# An Update on Molecular Mechanisms of Scarring—A Narrative Review

**DOI:** 10.3390/ijms252111579

**Published:** 2024-10-28

**Authors:** Michael Kohlhauser, Marcel Mayrhofer, Lars-Peter Kamolz, Christian Smolle

**Affiliations:** 1 Division of Plastic, Aesthetic and Reconstructive Surgery, Department of Surgery, Medical University of Graz, 8036 Graz, Austria; 2 COREMED—Centre for Regenerative Medicine and Precision Medicine, JOANNEUM RESEARCH Forschungsgesellschaft mbH, 8010 Graz, Austria

**Keywords:** scars, hypertrophic scars, keloid, atrophic scars, scarring, fibrosis, wound healing, healing process, fibroblasts, fibroblast heterogeneity

## Abstract

Fibroblasts, the principal cellular mediators of connective tissue remodeling, play a crucial role in the formation of physiological and pathological scars. Understanding the intricate interplay between fibroblasts and other cellular and molecular components is essential for elucidating the underlying mechanisms driving scar formation. Hypertrophic scars, keloids and atrophic scars arise from dysregulated wound healing processes characterized by persistent inflammation, aberrant collagen deposition, and impaired extracellular matrix remodeling. Fibroblasts play a central role in the pathogenesis of such pathological scars, driving aberrant extracellular matrix remodeling, subsequently contributing to the formation of raised or depressed fibrotic lesions. The investigation of complex interactions between fibroblasts and the microenvironment is crucial for developing targeted therapeutic interventions aimed at modulating fibroblast activity and improving clinical outcomes in patients with pathological scars. Further research into the molecular pathways governing fibroblast behavior and their heterogeneity holds promise for advancing scar management strategies. This narrative review was performed to shed light on the mechanisms behind scar formation, with a special focus on the role of fibroblasts in the formation of different types of scars, providing insights into the pathophysiology of these conditions. Through the analysis of current knowledge, this review seeks to identify the key cellular and molecular mechanisms involved in fibroblast activation, collagen synthesis, and extracellular matrix remodeling in hypertrophic scar, keloid, or atrophic scar formation.

## 1. Introduction

The skin is the largest organ of the human body and serves as the primary defense against harmful influences including physical trauma, microorganisms, or radiation. In addition to being able to register pressure, temperature and pain due to specific sensory receptors, it also regulates fluid loss, body hydration and temperature [1,2]. As in all mammals, human skin is composed of an epidermal layer, surrounding a stratified connective tissue, which consists of an upper papillary dermis, a deep reticular dermis, and the hypodermal subcutaneous tissue [3]. The physiological homeostasis of the body is greatly dependent on the integrity of human skin, which can be disturbed by disruption of the epidermis and dermis [4]. Any disruption that creates a wound is followed by a complex and dynamic cutaneous healing process, involving many cellular interactions and molecular mechanisms, in order to restore skin function and integrity. This process can be divided into four overlapping phases [2,5,6,7]. The first phase is hemostasis, which stops the bleeding after vascular damage [8], followed by inflammation, responsible for the defense against microorganisms [5]. The proliferative phase marks the third phase, consisting of neovascularization, granulation tissue formation, and re-epithelialization [5,6]. Finally, the remodeling phase occurs, which is responsible for scar tissue formation [5,6]. Even after the wound surface is completely covered, the remodeling phase persists for up to several months, leading to scar maturation, a process that can span up to two years [9]. Usually, a scar appears as a flat and pale patch of skin that is firmer and less flexible than the surrounding healthy tissue [9,10]. However, wound healing may be compromised by influences such as severe injuries, infection, and an extensive inflammatory response, leading to impaired skin repair and the formation of hypertrophic and keloid scars [11,12,13]. These pathological scars are associated with deep injuries to the dermis, which include burns, surgeries, lacerations, and abrasions [14,15]. Hypertrophic scars are raised, red, and rigid lesions confined to the original wound area, while keloids are overgrown and extend beyond the original injury site [10,16]. While hypertrophic scars develop rapidly within 6 months and regress only slowly over several years, there is no spontaneous regression observed in keloids [9]. Unlike these extensive scars, there is another type where the fibrosis is less pronounced, which are referred to as atrophic scars. Clinically, these scars occur as substance defects below the surface of the surrounding skin [17,18]. A clinical comparison of the different scar types is presented in Table 1. Fibroblasts are the primary type of dermal cells and play a crucial role in scar formation. In this review, we seek to elucidate the cellular and molecular mechanisms underlying various scar formations, with a particular emphasis on fibroblasts.

## 2. Physiological Scarring

Following skin injury, the immediate response involves platelet degranulation and the activation of both complement and clotting cascades. Due to this response, the formation of a fibrin clot occurs, which is crucial for hemostasis and also serves as a scaffold for subsequent cellular and molecular mechanisms [19]. Firstly, the degranulation of platelets results in the modulation of various cytokines, including platelet-derived growth factor (PDGF) and transforming growth factor beta (TGF-β), all of which in turn attract inflammatory cells [6,20]. Furthermore, molecules, namely, damage-associate molecular patterns (DAMPs), are released by stress cells undergoing necrosis. In the event that pathogens are also located in the wound, they will likewise release molecules, known as pathogen-associated molecular patterns (PAMPs). Both patterns are recognized by toll-like receptors (TLRs), thereby triggering intercellular signaling pathways of resident skin cells (e.g., dendritic cells, Langerhans cells, T cells, macrophages, and mast cells). These cells in turn not only lead to expression of cytokines but also chemokines, which subsequently attract inflammatory cells [6,21,22].

**Figure 1:**
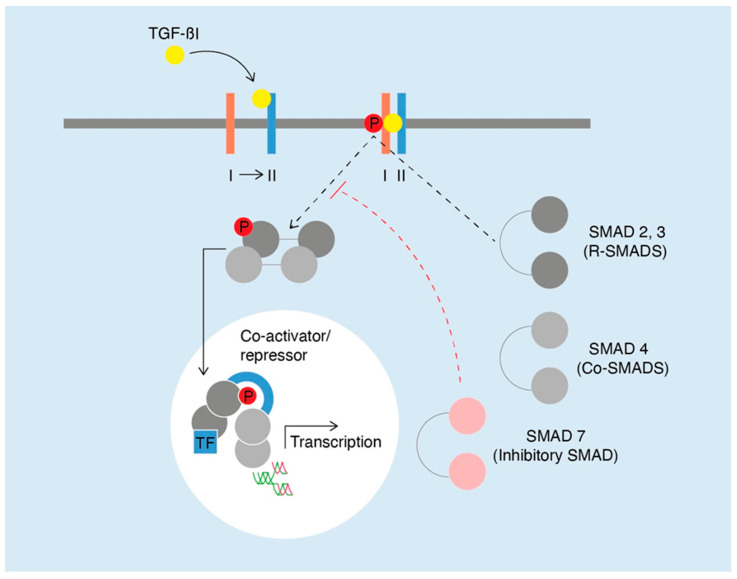
The SMAD signal-transduction pathway as a downstream mediator of TGF-β1 action: The TGF-β receptor consists of type I and type II subunits that are serine–threonine kinases, which signal through proteins of the SMAD family. The binding of TGF-β1 to its receptor type II leads to phosphorylation of the receptor type I and, consequently, to phosphorylation and activation of the R-SMAD proteins by receptor type I. Once activated, these SMADs form a complex with the common mediator Co-SMAD 4. This SMAD complex translocates into the nucleus, where the activated SMAD complex recruits other transcription factors (TFs) which, together, activate the expression of target genes mediating the biological effects of TGF-β1. Inhibitory SMAD 7 is able to prevent phosphorylation of R-SMADs via a negative feedback mechanism through the actions of TGF-β [39,40].

Leukocytes, especially neutrophil granulocytes, migrate into the wound, perform phagocytosis of bacteria, and secrete pro-inflammatory cytokines, such as tumor necrosis factor-alpha (TNF-α), interleukin (IL)-1β, and IL-6 [5,6]. Additionally, monocytes are recruited into the wound and differentiate under the influence of DAMPs and PAMPs into M1 macrophages. This macrophage subtype performs phagocytosis of microbes, neutrophils, and cellular debris and secretes pro-inflammatory mediators, such as IL-1, IL-6, IL-12, TNF-α, and nitric oxide [2,6]. Subsequently, M1 macrophages polarize into the M2 phenotype, which then secretes anti-inflammatory mediators such as IL-1R antagonist, decoy IL-1 receptor type II, IL-10, TGF-β, and the growth factors VEGF and IGF-1. These mediators and growth factors promote the transition to the proliferative phase [2,6]. This phase encompasses neovascularization characterized by restoration of the vascular network, the coverage of the wound surface through the formation of a new epithelial layer by keratinocytes, namely, re-epithelization, and the formation of granulation tissue, all of which occur simultaneously [2,5,6,7,8,23,24,25]. One of the predominant cells of the proliferative phase are fibroblasts, which are mainly responsible for the granulation tissue formation. Fibroblasts migrate to the wound in response to cytokines and growth factors, such as PDGF, TGF-β, and basic fibroblast growth factor (bFGF), produced by platelets and macrophages in the wound itself [6]. Inside the wound, fibroblasts degrade the fibrin-containing provisional matrix that emerges early in the hemostasis phase. Additionally, these cells deposit extracellular matrix (ECM) components, the majority of which include collagen, elastin, proteoglycans, and glycoproteins [5,6,26,27,28]. These then become the main components of the new granulation tissue, alongside the new blood vessels, macrophages, and fibroblasts themselves [2,5,6,26]. The resulting newly formed tissue fills the wound gap and provides a scaffold for cell adhesion, migration, growth, and differentiation during wound healing, thus enabling re-epithelialization [2,25,29]. During the formation of granulation tissue, the fibroblasts continue collagen synthesis [30], which provides structural support and strength to the new tissues. It plays a crucial role in wound healing by promoting tissue repair, wound closure, and eventually scar formation [5,6,26]. TGF-β1 is a multifunctional cytokine produced by various cells (e.g., macrophages, endothelial cells, keratinocytes, fibroblasts) during the wound healing process [31]. Several studies suggest that the synthesis of collagen and other ECM components in fibroblasts is triggered by TGF-β1 [32,33,34]. As with all TGF-β family ligands, TGF-β1 initiates biological effects via the TGF-β1/Smad pathway [10,32,35]. A brief description of the TGF-β1/SMAD pathway is displayed in Figure 1. A downstream mediator induced by TGF-β1 is connective tissue growth factor (CTGF), which plays an important role in regulating fibroblast function and stimulates the production of ECM components [36]. Additionally, IL-6 and PDGF have the ability to stimulate the production of different collagens in dermal fibroblasts and also to increase their synthesis of TGF-β1 [37,38].

**Figure 2:**
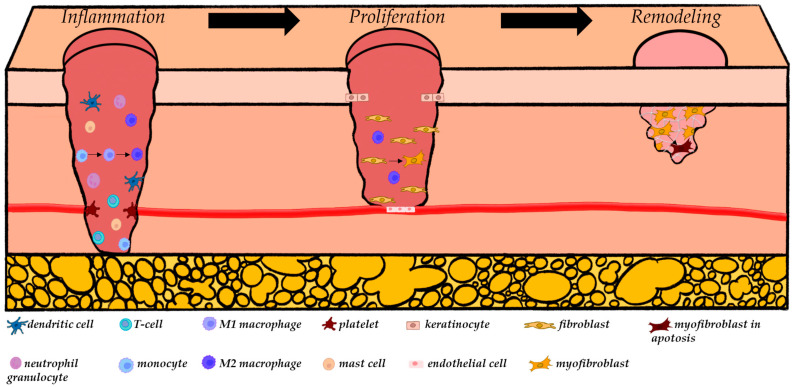
A brief summary of the wound healing process with a focus on scar formation. Following an injury, the formation of a fibrin clot occurs due to platelet degranulation, which is crucial for hemostasis. Additionally, the platelet degranulation leads to an increase in TGF-β1 and PDGF, which subsequently results in an influx of inflammatory cells (e.g., dendritic cells, Langerhans cells, T cells, leucocytes, macrophages, and mast cells). These cells release pro-inflammatory cytokines. Leukocytes and M1 macrophages, which are derived from monocytes, perform phagocytosis of harmful microorganisms and cell debris. After phagocytosis, M1 macrophages polarize into the M2-subtype, which secrete anti-inflammatory cytokines (e.g., IL-1Ra, IL-10, VEGF, IGF-1, TGF-β1, and PDGF), initiating the shift towards the proliferative phase. The presence of these cytokines and growth factors induces the migration of various cell types, including keratinocytes, endothelial cells, and fibroblasts. While keratinocytes are involved in re-epithelization and endothelial cells in re-vascularization, fibroblasts are responsible for the formation of granulation tissue. To facilitate this process, fibroblasts synthesis ECM components, which, in conjunction with macrophages and the newly formed vessels, contribute to the formation of granulation tissue. The synthesis of ECM is modulated by growth factors, specifically TGF-β1 and PDGF. As the process progresses, fibroblasts differentiate into myofibroblasts, which are of great importance for the contraction of the wound and undergo apoptosis after finishing the remodeling phase. Meanwhile, the production of ECM continues. Concurrently, the degradation of ECM occurs via MMPs, which are regulated by TIMPs, whose synthesis is induced by MMPs themselves. This establishes a balance between the synthesis and degradation of ECM during scar remodeling.

Through mechanical tension and the influence of distinct growth factors, primarily TGF-β1, fibroblasts transdifferentiate into myofibroblasts that are essential for wound contraction due to the expression of contractile alpha-smooth muscle actin (α-SMA) [41,42,43]. Myofibroblasts are characterized by an increased secretion of ECM components and play a crucial role in scar maturation [44,45,46]. In physiological scar formation, myofibroblasts usually undergo apoptosis after completing the remodeling process, resulting in a less cellular scar [41,47]. Throughout the remodeling process, matrix metalloproteinases (MMPs) play a crucial role in minimizing scarring by the proteolytic destruction of ECM components [48,49,50,51]. In human biological research, 23 unique isoforms of MMPs have been identified [52,53,54], among which the collagenases MMP-1, -8, and -13 cleave interstitial collagens I, II, and III, while the gelatinases MMP-2 and -9 digest denatured collagens [55]. Subsequently, the initial collagen III is progressively replaced by mature collagen I, which has more tensile strength [56,57] and is organized into small parallel bundles, thus differing from the basket-weave collagen in uninjured dermis [58]. During remodeling, it is of utmost importance to maintain the balance between the deposition and degradation of the synthesized collagen [10]. Insufficient collagen synthesis may inhibit wound closure and tissue repair, while excessive collagen production expedites pathological scar formation [10,59,60,61]. To achieve this balance, MMPs stimulate their suppressors, known as tissue inhibitors of metalloproteinases (TIMPs) [62,63,64]. Fibroblasts are also crucial in the synthesis of MMPs and TIMPs at this stage [65]. Figure 2 presents a schematic summary of the cellular composition during the different phases of physiological wound healing, highlighting the cells involved in the formation of a physiological scar.

## 3. Dermal Fibroblast Heterogeneity

Despite the presence of fibroblasts in all connective tissues, significant advances have been made in understanding their specific role within the dermis. As previously mentioned, dermal fibroblasts maintain skin integrity and homeostasis by producing ECM components and remodeling enzymes and by releasing paracrine and autocrine signals that modulate ECM production, cellular states, and signaling pathways during wound healing [27,66,67,68].

Although fibroblasts are frequently depicted as a homogenous cellular entity in scholarly discourse, it has been determined that various types of fibroblasts exist, occupying distinct layers of the skin [67,69,70,71,72]. The first distinction among dermal fibroblasts is observed in their embryonic origins. Studies in mice have shown that craniofacial fibroblasts derive from the neural crest, while those in the dorsal region originate from the dermomyotome. In contrast, dermal fibroblasts of the ventral and flank trunk, as well as the limb dermis, originate from the lateral plate mesoderm [73,74,75]. Another variance is not confined to the location but encompasses their morphology and cellular properties. Recent investigations showed that fibroblasts of the distinct human skin layers demonstrate different morphological characteristics. Papillary fibroblasts possess a lean, spindle-shaped morphology, while reticular fibroblasts have a square and stretched one [65,76]. Furthermore, it has been shown that fibroblasts from the papillary layer have a higher proliferative capacity and a longer life-span in culture, compared to those in the reticular dermis [76,77,78]. Another distinction is evident in the production of proteoglycans; while papillary fibroblasts secrete more decorin, those in the reticular layer focus on versican production [79,80]. Bahar et al. [81] illustrated that fibroblasts isolated from distinct dermal layers demonstrate the differential expression of ECM components. These include type I and III procollagen and collagenases, with a marked increase in collagenase mRNA in fibroblasts from the papillary dermis. Conversely, less collagenase production was found in human reticular fibroblasts, along with higher α-SMA gene expression and collagen synthesis [76,82,83]. Furthermore, Haydont et al. [84] identified particular fibroblasts from the dermo-hypodermal junction of human skin, associated with the organization and remodeling of the ECM. This corresponds with the findings of Driskell et al. [85], who demonstrated in a mouse model that fibroblasts from the reticular and hypodermal layer play a major role in the repair of the dermis. Skin fibroblast heterogeneity is not limited to papillary and reticular fibroblasts but extends to different cells populating the various layers of the skin. An analysis of explant cultures derived from these different dermal layers has shown variances in fibroblast behavior in culture and in gene expression [76,86,87]. In recent years, several rodent studies using single-cell RNA sequencing (scRNA-seq) have enabled the characterization of the transcriptional heterogeneity of dermal fibroblasts [88,89,90,91,92]. However, to date, only a few studies have concentrated on the identification of distinct fibroblast subpopulations within human skin [93,94,95,96,97]. In a study by Tabib et al. [93], two different major types of dermal fibroblasts are distinguished. The predominant fibroblast subgroup is characterized by the co-expression of secreted frizzled-related protein 2 (SFRP2) and dipeptidylpeptidase 4 (CD26). These types of fibroblasts are marked as extended bipolar cells, which are primarily located within collagen bundles and demonstrate increased expression of ECM components, including collagen I, fibrillin, and fibronectin. Alternatively, another subgroup, which has a larger cell volume and nuclei compared to SFRP2 + CD26+ fibroblasts, shows flavin-containing mono-oxygenase 1 (FMO1) and lymphocyte-specific protein 1 (LSP1) markers and expresses lower levels of COL1A1 and COL1A2. In addition, the authors found five minor fibroblast populations. In contrast, Solé-Boldo et al. [96] propose a division into four major fibroblast subpopulations that can be spatially localized and exhibit distinct functional annotations, including secretory-papillary, secretory-reticular, mesenchymal, and pro-inflammatory fibroblasts. Although secretory-papillary and -reticular fibroblasts are located in different layers of the dermis, these subpopulations exhibit a strong enrichment of classical fibroblast functions, such as collagen production and ECM organization. Notwithstanding their location in the reticular dermis, the inflammatory fibroblasts are minimally involved in ECM production and organization while exhibiting strong pro-inflammatory functions, including regulating inflammatory responses and chemotaxis. In contrast, the mesenchymal subtype exhibits enhanced mesenchymal potential, contributing more to cartilage and bone development, with fibroblasts expressing specific collagens COL11A1 and COL24A1, and are primarily located in the reticular dermis. Similar results were reported by Deng et al. [97]. Korosec et al. [95] identified cell surface markers for papillary (FAP + CD90-) and reticular (FAP + CD90+, FAP-CD90+) fibroblasts in human skin. FAP + CD90- papillary fibroblasts express podoplanin (PDPN), netrin 1 (NTN1), and high amounts of CD26 and CD39. The reticular fibroblasts express α-SMA, matrix gla protein (MGP), peroxisome proliferator-activated receptor gamma (PPARγ), and more CD36. Philippeos et al. [94] identified five distinct subtypes of fibroblasts, each exhibiting unique surface markers. The first population was CD90 + CD39 + CD26-, characterized by the expression of specific collagen chains, such as COL6A5, and is localized in the upper dermis. The other populations are situated in the reticular dermis, either marked by CD90 + CD36 + PPARγ+, CD90 + CD39-RGS5+, CD90 + CD39 + CD26+, or CD90 + CD39-RGS5-. In summary, all three studies have identified CD26 as a key surface marker for a subpopulation of human skin fibroblasts. Worthen et al. [98] suggests that CD26+ fibroblasts are the primary producers of collagen during human wound healing, noting that these cells double following injury and account for 85 percent of COL1A1 gene expression. Increased collagen production by CD26+ fibroblasts has also been observed in animal studies involving mice and red duroc pigs [99,100]. Recent studies using scRNA-seq of human dermal fibroblasts demonstrated that fibroblast function changes with age. The expression of key ECM components, elastin-related genes, and collagen family genes was negatively impacted by age, while the levels of MMP secretion and the expression of genes involved in inflammation, lipid metabolism, and adipogenesis were elevated [96,101]. The loss of ECM components may be the reason for wrinkles and loss of elasticity in elderly skin, as well as its reduced wound healing capacity.

## 4. Hypertrophic Scarring

Hypertrophic scars may arise as a result of dysfunction of the healing process and are associated with deep injuries to the dermis such as burns, surgeries, lacerations, and abrasions [10]. In burns in particular, the prevalence of hypertrophic scars is reported to be up to 72 percent [9]. According to the current literature, it is assumed that the formation of hypertrophic scars is associated with pathologically increased inflammation and a prolonged healing process linked to necrosis and infection [37,39,40,41]. These factors in turn lead to the presence of increased levels of PAMPs, DAMPs, and TLR signaling in the wound itself. The subsequent result is a pronounced infiltration of inflammatory cells, which in turn leads to an augmented inflammatory response [11,13,102,103,104,105]. Neutrophils and T-cells have been observed in significant numbers in hypertrophic scars [106,107]. In addition, hypertrophic scarring is associated with an increased number of macrophages, with an initial dominance of type M1, which polarize to the M2 subtype at a deferred time point [104,108,109]. Neutrophils, T-cells, and M1 macrophages release cytokines such as IL-1β, IL-6, and TNF-α, while M2 macrophages secrete growth factors such as TGF-β1 and PDGF [110,111,112,113]. IL-1β and TNF-α induce the expression of chemokines that attract immune cells and enhance cytokine action [114,115,116]. Within this intensified inflammation, an up-regulation of TGF-β1 and PDGF also occurs. Both TGF-β1 and PDGF are important profibrotic growth factors, which enhance fibroblast proliferation, induce their differentiation into myofibroblasts, and stimulate their collagen production [6,13,37]. Augmented levels of TGF-β1 and PDGF are associated with hypertrophic scar formation [117,118,119]. In addition to inflammatory cells, fibroblasts also play a pivotal role in this augmentation, as they have the capacity to induce their TGF-β1 production autonomously [120], and this synthesis is upregulated in the formation of hypertrophic scars [117,121]. Furthermore, PDGF has been found to increase in both the epidermis and the dermis of hypertrophic scars [122]. Different members of the PDGF family have been reported to stimulate the expression of TGF-β receptors and thus increase TGF-β1 secretion in fibroblasts [37,123,124,125]. According to a study by Wang et al., the majority (72.7%) of TGF-β1 production appears to originate from the fibroblasts of the deep reticular dermis, and a substantial part of myofibroblasts are derived from these cells [83]. Another cytokine that is able to up-regulate the production of TGF-β1 is IL-6, which is also capable of enhancing the rate of fibroblast differentiation and their expression of collagen. Fibroblasts, in turn have an added ability to secrete IL-6 to enhance this purpose [37,106]. In a recent study, Huang et al. [126] demonstrated that the levels of CD39+ fibroblasts, located in the papillary dermis, were elevated in hypertrophic scars and positively correlated with the severity of the disease. The authors suggest that CD39+ fibroblasts synthesize important profibrotic cytokines, including IL-11, which trigger the underlying α-SMA-positive myofibroblasts to produce ECM. IL-11 is a relatively unknown member of the IL-6 family. It was found to have ERK-dependent pro-fibrotic activity in fibroblasts. In a recent study, Adami et al. [127] suggest that IL-11 facilitates the transformation of fibroblasts into myofibroblasts via ERK activation and enhances ECM synthesis. In addition, an increased number of mast cells accumulates during hypertrophic scar formation [128,129], and several individual studies intimate that this augmentation is pivotal in this process. Chen et al. indicated that mast cell chymase stimulates TGF-β1 action via the TGF-β1/Smad signaling pathway [130]. Fibroblast migration and proliferation are also promoted through mast cells mediators such as histamine and tryptase [131,132,133]. Artuc et al. suggest that mast cells promote fibroblast proliferation by inducing fibroblast growth factor (FGF)-7 and bFGF secretion from fibroblasts mediated by histamine and tryptase [134]. Additionally, these two mediators are thought to be associated with fibroblast differentiation into myofibroblasts [135].

Contrary to physiological scarring, the apoptosis of myofibroblasts fails in hypertrophic scar formation [6,26,41,136], thus resulting in an augmented level, which in turn leads to elevated concentrations of α-SMA and an extensive ECM through an enriched collagen synthesis [10,43]. In these scars, the collagen synthesis is estimated to be seven-times higher than in uninjured healthy skin [62]. Compared with physiological scarring, the synthesis of collagen III in hypertrophic scars is upregulated [137]. Additionally, the progression of hypertrophic scar formation is characterized by an imbalance between ECM synthesis and degradation [55], caused by a reduction in the expression of MMP-1, along with increased levels of TIMP-1 [48,62]. This imbalance results in excessive collagen deposition [10,41,57,59,60]. The collagen bundles in hypertrophic scars, which are predominantly of type III, feature a nodular morphology and are organized in a parallel alignment to the epidermis, unlike the typical basket weave-like structure seen in uninjured skin [10,138,139].

In addition to collagen, other constituents of the ECM, such as proteoglycans and elastin, exhibit alterations in hypertrophic scars. The expression of proteoglycans, decorin, and fibromodulin are lower in fibroblasts derived from hypertrophic scars compared with those from uninjured dermis [140,141,142]. Both decorin and fibromodulin are small leucine-rich proteoglycans and are able to modulate TGF-beta activity [143]. The two proteoglycans each decrease the TGF-β1-induced expression of plasminogen activator inhibitor-1 (PAI-1) [144,145], a molecule that facilitates the excessive accumulation of collagen [146]. Furthermore, decorin has been reported to attenuate some TGF-β actions, including fibroblast proliferation, myofibroblast trans-differentiation and TGF-β1 production by fibroblasts [144,147]. Recent findings suggest that fibromodulin supports the early migration of fibroblasts and their trans-differentiation into myofibroblasts through the TGF-β1/Smad pathway. Additionally, it inhibits TGF-β1 expression and auto-induction, facilitating smaller and more tensile scar tissue with no extensive ECM deposition [145]. Interestingly, several studies have demonstrated that fibroblasts isolated from the reticular dermis synthesize less decorin and fibromodulin than those from the papillary dermis [83,141,142,147]. Conversely, the expression of versican, a large proteoglycan, is enhanced in reticular fibroblasts [83]. In hypertrophic scars, versican is six-fold higher and is co-responsible for scar rigidity through its strong hydrophilic properties [148,149].

**Figure 3:**
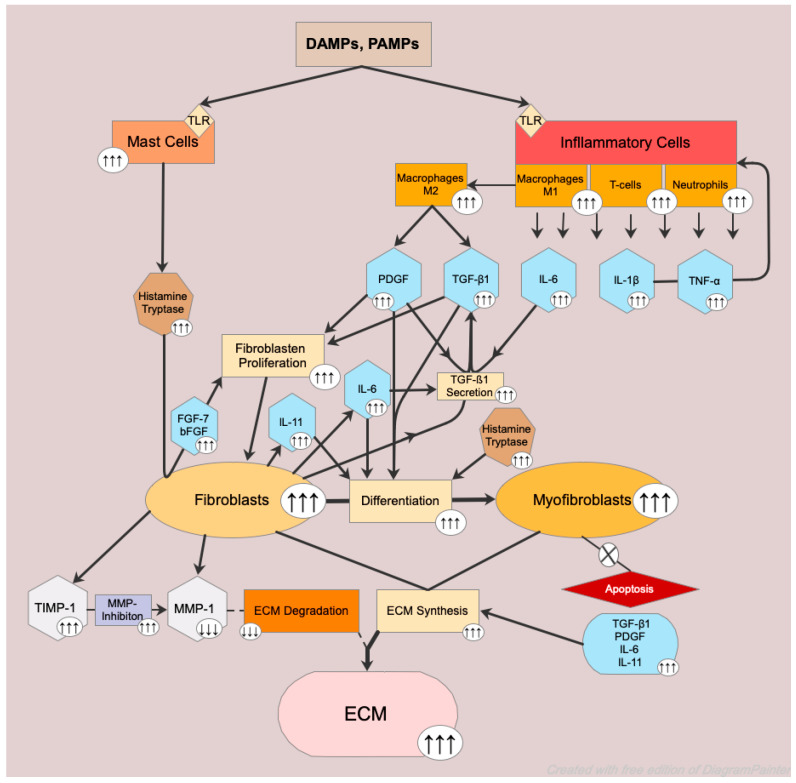
Schematic illustration of the above-mentioned molecular mechanism of hypertrophic scarring. DAMPs and PAMPs activate via TLR inflammatory cells, which induces the secretion of TGF-β1 and PDGF, as well as the pro-inflammatory cytokine IL-6. TGF-β1 and PDGF increase fibroblast proliferation, while PDGF and IL-6 further enhance the secretion of TGF-β1, which consequently leads to elevated fibroblast proliferation and the activation of its cellular functions. Simultaneously, DAMPs and PAMPs also activate mast cells via TLR, which promote the secretion of bFGF and FGF-7 from fibroblasts through histamine and tryptase. In turn, these growth factors are able to increase fibroblast proliferation. Subsequently, the differentiation of fibroblasts to myofibroblasts is facilitated by PDGF, TGF-β1, IL-6, and IL-11. The upregulated fibroblast differentiation and absence of apoptosis of the myofibroblasts result in the accumulation of the latter. The high volume of PDGF, TGF-β1, IL-6, and IL-11 results in an increase in ECM synthesis. Increased TIMP-1 inhibits MMP-1 actions, thus causing downregulated ECM degradation, which ultimately leads to extensive ECM deposition.

Elastic fibers are extracellular matrix macromolecules, which are essential for the elasticity of the tissue, and are composed of elastin surrounded by fibrillin [150,151]. A study by Bhangoo et al. showed that elastic fibers in hypertrophic scars exhibited a disturbed arrangement, predominantly localized in the papillary layer, with an uneven distribution and notable absence in some reticular regions [152]. Immunohistochemical analyses of its main components demonstrated disturbed fibrillin arrangement in the papillary dermis with no candelabra-like configuration, typically in uninjured skin. In the reticular dermis, fibrillin was fragmented, and elastin was found exclusively in certain deposits [153,154]. Furthermore, both exhibited a reduced volume in hypertrophic scars compared to uninjured skin and physiological scars [154]. A schematic illustration of the molecular mechanism of hypertrophic scarring is illustrated in Figure 3.

## 5. Keloid Scarring

The first records of extensive scarring date back to as early as 1700 BC [155]. Throughout the last 60 years, there has been an increasing differentiation between hypertrophic scars and the formation of keloids. Although hypertrophic scars and keloids share some characteristics, there are also inherent differences. Both types of scars extend above the usual skin level, but keloids also extend beyond the area of the initial wound surface and can be differentiated by clinical, histological, and epidemiological aspects [156,157]. For keloid formation, minimal injuries are often enough to lead to excessive scarring over time. Even the spontaneous formation of keloids occurring without any known trauma or injury has been extensively described [158]. Histologically, the scar tissue of keloids contains an excess of dermal collagen, especially disorganized type I and III. The pale-colored hypocellular collagen occurs in bundles and without nodules or excess myofibroblasts [57]. Similar to hypertrophic scars, keloids show an excessive presence of fibroblast proteins, which indicates the prolonged persistence of wound healing signals [159].

Several studies suggest hormones play an important role in keloid formation. This would be also be suggested by the increased occurrence of keloids in pregnant and pubertal individuals [160,161].

As already mentioned above, the transformation of a wound clot into granulation tissue requires a delicate balance between the deposition and degradation of extracellular matrix proteins. When this process is disrupted, abnormalities in scar formation occur, leading to the formation of either keloids or hypertrophic scars [148,162]. Recent evidence strongly suggests that a prolonged period of inflammation in which immune cells infiltrate is present in the scar tissue of keloids, which may lead to increased fibroblast activity. Elucidating this process could help explain why keloid scars spread beyond the area of the initial wound, while hypertrophic scars, with their decrease in immune cell infiltration over time, remain within the initial wound borders and often regress over time [162]. A significant contrast to hypertrophic scars is that in keloids, myofibroblasts undergo apoptosis, but the proliferation rate of fibroblasts is markedly elevated, resulting in an abnormal accumulation of these cells [163]. CD26+ fibroblasts have been reported to exhibit higher proliferation rates compared to CD26- ones [164]. This could potentially elucidate the pathomechanism behind the emergence of keloids, typified by aberrant fibroblast proliferation. Affirmation of this assumption would align with the findings of Xin et al. [165], indicating that fibroblasts from keloids exhibited higher expression levels of CD26 compared to those from normal scars.

In recent years, more evidence has been collected demonstrating that it is not just the strength and length of the inflammatory reaction that matters but also the type of immune response. The characteristic cytokine expression profile of CD41 cells provides the basis for describing either a predominantly T-helper (TH)-1 or Th-2 immune response [148,166]. While an increased Th-1 response appears to attenuate fibrosis via interferon y and interleukin 12, a primary Th-2 response via IL-4, IL-5, IL-10, and IL-13 is associated with excessive tissue fibrosis [166]. These mediators subsequently drive processes such as the recruitment, proliferation, and matrix deposition of fibroblasts; angiogenesis; and re-epithelialization. Fibroblast activity and the inhibition of protease production, which are essentially necessary to maintain the balance between production and degradation, are mainly controlled by the fibrogenic growth factors PDGF, IGF-I, FGF-β, and TGF-β [10,167].

However, the inflammatory reaction alone does not seem to be responsible for keloid formation, as multiple studies did not show a significant improvement in the case of fibroproliferative diseases, even though immunomodulatory therapies were administered [168]. Central to the formation of keloid scar tissue is a change in the fibroblast phenotype. Keloid-derived fibroblasts (KDFs) show distinct gene expression profiles compared to normal fibroblasts. They often have increased expression of genes associated with fibrosis, such as collagen I, collagen III, and fibronectin, which contributes to excessive ECM production. In addition, KDFs have reduced expression of MMPs, which normally support ECM degradation, leading to an imbalance that favors fibrosis. Keloid fibroblasts have increased numbers of growth factor receptors and respond more rapidly to growth factors such as PDGF and TGF-β, which may upregulate these abnormal cells from the onset of wound healing [169,170,171,172]. When investigating critical pathways for the formation of keloids, studies found increased expression of several proteins related to IGF and IGF binding. A reduced expression of a subset of Wnt signaling inhibitors and several IL-1-inducible genes has also been described. These findings suggest epigenetic silencing of a subset of genes in the altered fibroblasts of keloids [173].

TGF-β has been linked to keloid formation in various ways. Overexpression of TGF-β1 and -β2 was found in keloids and keloid-derived fibroblasts, with significantly lower TGF-β3 expression [174,175,176,177].

Using single-cell sequencing, researchers have identified distinct fibroblast populations in the human dermis on the basis of their transcriptional profile [96,97,178,179,180]. Serror et al. [180] were able to identify two keloid entities through fibroblast features. Even within keloids, fibroblasts were found to differ according to their location in the center or periphery of the wound and the superficial or deep dermis. Differences between these subpopulations of fibroblasts, including papillary and reticular cells as well as central and peripheral fibroblasts, have been validated. Solé-Boldo et al. [96] identified four subpopulations of human dermal fibroblasts, including secretory-papillary, secretory-reticular, pro-inflammatory, and mesenchymal fibroblasts. Recent studies have shown that the latter ones were identified as the fibroblasts that contribute most to keloid formation [97,179]. A crucial marker for mesenchymal fibroblasts is periostin (POSTN), which is observed at high levels in keloids [97,179,181]. In a study by Deng et al. [97], increased levels of endothelial cells and smooth muscle cells in keloids were found, which is consistent with further studies that demonstrated increased angiogenesis in keloids [182,183]. Recently, Liu et al. [178] suggested that activation of TGF-β signaling in endothelial cells has a role in the extensive angiogenesis of keloids. Zhou et al. [184] illustrated that POSTN expression levels could be regulated by TGF-β in human dermal fibroblasts, and thus, TGF-β could initiate the mesenchymal activation of endothelial cells. Interestingly, Shim et al. [181] described the increased expression of POSTN in both mesenchymal fibroblasts and endothelial cells in human keloid tissue samples. To validate the observed findings, the authors conducted further analysis of the data from keloid endothelial cells and affirmed the overexpression of SMADs, which are downstream mediators of TGF-β. Consequently, it can be inferred that dysregulated TGF-β/SMAD signaling contributes to keloid formation by promoting not only extensive fibrosis but possibly also neovascularization. Transcriptional profiling of fibroblasts revealed 834 differentially expressed genes between nodular and extensive keloids. Quantitative analysis of ECM-related gene expression using RT-qPCR showed that central reticular fibroblasts in nodular keloids produce higher levels of mature collagens, TGFβ, HIF1α, and αSMA compared to control skin [180]. This suggests that the central deep region of nodular keloids is the primary site of ECM production, extending centrifugally in keloids. While no significant differences in basal proliferation were observed, peripheral fibroblasts from extensive keloids demonstrated greater migration than both central fibroblasts and nodular cells. Additionally, these peripheral fibroblasts from extensive keloids exhibited stronger traction forces than central cells, control fibroblasts, and nodular fibroblasts [180].

## 6. Atrophic Scars

In contrast to hypertrophic scars and keloids, atrophic scar formation results in less fibrosis, leading to depressed scars below the level of the surrounding skin [17,18]. These scars can be further divided into subtypes, including ice pick scars, which make up 60 to 70%; boxcar scars, which account for 20 to 30%; and rolling scars, which comprise 15 to 25% [185,186]. The formation of atrophic scars is particularly associated with acne, with 80 to 90% of all acne scars being atrophic [186]. A study from 1994 by Layton et al. [187] demonstrated that in 95% of cases, acne patients developed scars, with 30% experiencing severe forms. As with other pathological scars, inflammation plays a key role in the development of atrophic ones. An initial weak but persistent inflammatory response, without downregulation and with gradual increase, was observed in acne patients who developed atrophic scars [188,189]. Propionibacterium acnes induces the innate immune response, primarily via TLRs, stimulating the expression of the pro-inflammatory cytokines IL-1, IL-6, IL-8, IL-12, and TNF-α [190,191,192,193,194,195]. Significantly increased levels of IL-1β (16-fold), TNF-α (2.6-fold), and IL-8 (3015-fold) were found in acne lesions compared to healthy skin [196]. IL-1β is known as a potent inducer of both IL-6 and IL-8 [195]. IL-8 acts as a potent chemoattractant for neutrophils, which release lysosomal enzymes, subsequently leading to the rupture of the follicular epithelium and further inflammation [197]. In contrast, IL-12 promotes Th1-differentiation and their IFN-γ production via signal transducer and activator of transcription 4 (STAT4) phosphorylation [198,199,200]. IFN-γ is considered as antifibrotic because it inhibits collagen synthesis in fibroblasts [201,202] In the course of this inflammatory stage, a loss of dermal matrix in the form of collagen breakdown is observed, which is typical of atrophic scars [203]. Furthermore, IL-10 was found to be significantly increased in acne lesions compared to healthy skin [196]. IL-10 is known as an antifibrotic cytokine and acts as a crucial regulator of fibrotic processes [204]. Histologically, the following changes can be observed in atrophic scars: absence of hair follicles and sebaceous glands, decreased epidermal thickness, increased cellularity including fibroblasts and myofibroblasts, abnormal collagen fiber density and orientation with partially loose arrangement, and mild inflammatory infiltration [205]. Atrophic scars are characterized by less collagen [206] and fewer elastic fibers than healthy skin [207]. TNF-α, an important modulator of MMP activity in the dermis [208,209], was found to have a five-fold increase in acne lesions compared to healthy skin [196]. Increased levels of MMP-1, MMP-2, MMP-3, MMP-9, and MMP-12 have been shown to be present in acne lesions [196,210,211]. A recent study demonstrated that during atrophic scar development, elevations in MMP-1, MMP-2, and MMP-12 occur, accompanied by a simultaneous reduction in TIMP-1 and TIMP-2. These consequently leads to lower levels of elastic fibers, as well as collagen 1 and 2 [212].

Vimentin is a cytoskeletal protein, which is important for fibroblast function [213]. In a mouse model, excessive vimentin degradation led to reduced fibroblast activity, resulting in inhibited fibrosis, which may be a reason for the development of atrophic scars [214].

## 7. Conclusions

In conclusion, fibroblasts serve as central orchestrators in both physiological wound healing and the pathogenesis of hypertrophic scars and keloids. In physiological wound healing, fibroblasts play a crucial role in ECM deposition, remodeling, and wound contraction, facilitating tissue repair and the restoration of tissue integrity. However, the dysregulation of fibroblast activity leads to the formation of hypertrophic scars and keloids, characterized by excessive collagen deposition and aberrant ECM remodeling. Fibroblasts within scar tissue exhibit phenotypic changes, including increased proliferation, collagen synthesis, and differentiation into myofibroblasts, perpetuating the fibrotic phenotype. Numerous molecular mediators, such as TGF-β, PDGF, and FGF, intricately regulate fibroblast behavior and contribute to scar pathogenesis. Understanding the complex interplay between fibroblasts and the microenvironment is essential for elucidating the underlying mechanisms driving scar formation and identifying potential therapeutic targets. Targeted interventions aimed at modulating fibroblast activity and restoring ECM homeostasis hold promise for improving clinical outcomes in patients with hypertrophic scars and keloids. Further research into the molecular pathways governing fibroblast behavior is warranted to advance scar management strategies and enhance our understanding of wound healing processes. By unraveling the intricate role of fibroblasts, we can pave the way for more effective treatments and interventions to address the challenges posed by hypertrophic scars and keloids.

Understanding the heterogeneity of fibroblasts in pathological wound healing and scarring opens new possibilities for therapeutic interventions. Current treatments such as corticosteroid injections, surgical excision, and laser therapy often produce inconsistent results, partly linked in several studies to the complex nature and heterogeneity of fibroblasts. Targeting specific fibroblast subpopulations or their signaling pathways could lead to more effective and personalized treatments.

## Figures and Tables

**Table 1:** This table is intended to provide a brief and concise overview of the characteristics of the presented types of scars.

	PHYSIOLOGICAL SCAR	HYPERTROPHIC SCAR	KELOID	ATROPHIC SCAR
CLINICAL FEATURES	▪ Flat and pale skin patch ▪ At the level of the surrounding skin ▪ Firmer and less flexible than healthy tissue	▪ Red and rigid lesion ▪ Rises above skin level ▪ Does not extend beyond initial site of injury	▪ Red and rigid lesion ▪ Rises above skin level ▪ Typically projects beyond the original wound margins	▪ Sunken or pitted areas
TIMEFRAME	▪ Scar maturation lasts for several months, up to two years	▪ 4–8 weeks after injury ▪ Rapid growth phase for up to 6 months ▪ Gradually regresses over years	▪ Develops over years ▪ No spontaneous regression	▪ Weeks to months after the initial injury
HISTOLOGY	▪ Collagen fibers are realigned, cross-linked, and converted from type III to type I collagen, which has more tensile strength ▪ The collagen is organized in small parallel bundles, in contrast to the basket-shape in uninjured dermis	▪ Primarily type III collagen ▪ Oriented parallel to the epidermal surface ▪ Presence of parallel arrays of thick collagen bundles ▪ Inflammatory infiltrate comprising lymphocytes and macrophages	▪ Type I and III collagen ▪ Disorganized collagen bundles ▪ Abundant fibroblasts arranged in nodules throughout the dermis ▪ Absence of inflammatory infiltrate ▪ Altered ECM composition with increased glycosaminoglycans	▪ Loss of collagen, elastin, and other extracellular matrix components ▪ Thinner dermis and epidermis
PHYSIOLOGY/ PATHOPHYSIOLOGY	▪ Fibroblasts transdifferentiate into myofibroblasts ▪ Balanced ECM synthesis and degradation via MMP and TIMP interplay ▪ Apoptosis of myofibroblasts, resulting in a less cellular scar	▪ Excessive inflammatory response with increased cytokine and growth factor release ▪ Absence of myofibroblast apoptosis ▪ Excessive synthesis of collagen and other ECM components ▪ Imbalance between ECM synthesis and degradation	▪ Dysregulated fibroblast proliferation and collagen synthesis ▪ Enhanced expression of pro-inflammatory cytokines ▪ Genetic predisposition and altered signaling pathways (e.g., MAPK, NF-κB)	▪ Collagen deficiency, often due to impaired fibroblast function ▪ Enhanced degradation of the extracellular matrix ▪ Prolonged inflammation or infection can exacerbate tissue loss

## Data Availability

Due to the nature of this work, no new data were generated. The data can be obtained from the respective original paper.

## Abbreviations

PDGF	Platelet-derived growth factor
TGF	Tissue growth factor
DAMPs	Damage-associate molecular patterns
PAMPs	Pathogen-associated molecular patterns
TLR	Toll-like receptor
TNF-α	Tumor necrosis factor-alpha
IL	Interleukin
bFGF	Basic fibroblast growth factor
ECM	Extracellular matrix
CTGF	connective tissue growth factor
TF	transcription factors
α-SMA	Alpha-smooth muscle actin
MMP	Matrix metalloproteinase
TIMP	Tissue inhibitor of metalloproteinases
SFRP 2	Secreted frizzled-related protein 2
CD	Dipeptidylpeptidase
FMO1	Flavin-containing mono-oxygenase 1
LSP1	Lymphocyte-specific protein 1
COL1A	Collagen type I alpha
FAP	Familial adenomatous polyposis
PDPN	Podoplanin
NTN1	Netrin 1
MGP	Matrix gla protein
PPARγ	Peroxisome proliferator-activated receptor gamma
FGF	Fibroblast growth factor
PAI-1	Plasminogen activator inhibitor-1
Th	T-helper
KDFs	Keloid-derived fibroblasts
POSTN	Periostin
STAT4	Signal transducer and activator of transcription 4
ScRNA-seq	Single-cell RNA sequencing
SFRP2	Secreted frizzled-related protein 2
CD26	Dipeptidylpeptidase 4
FMO1	Flavin-containing mono-oxygenase 1
LSP1	Lymphocyte-specific protein 1
PDPN	Podoplanin
NTN1	Netrin 1
